# *EbbHLH80* Enhances Salt Responses by Up-Regulating Flavonoid Accumulation and Modulating ROS Levels

**DOI:** 10.3390/ijms241311080

**Published:** 2023-07-04

**Authors:** Qingqing Gao, Xia Li, Chunfan Xiang, Ruolan Li, Hongchun Xie, Jia Liu, Xiaoning Li, Guanghui Zhang, Shengchao Yang, Yanli Liang, Chenxi Zhai, Yan Zhao

**Affiliations:** 1 National-Local Joint Engineering Research Center on Gemplasm Innovation & Utilization of Chinese Medicinal Materials in Southwest, The Key Laboratory of Medicinal Plant Biology of Yunnan Province, Yunnan Agricultural University, Kunming 650201, Chinaliangyanlimt@sina.com (Y.L.); 2 College of Agronomy & Biotechnology, Yunnan Agricultural University, Kunming 650201, China; 3 Sibley School of Mechanical and Aerospace Engineering, Cornell University, Ithaca, NY 14850, USA

**Keywords:** *EbbHLH80*, salt tolerance, flavonoids, ROS, stress-responsive genes, *Erigeron breviscapus*

## Abstract

*bHLH* transcription factors are involved in multiple aspects of plant biology, such as the response to abiotic stress. *Erigeron breviscapus* is a composite plant, and its rich flavonoids have strong preventive and therapeutic effects on cardio cerebral vascular disease. *EbbHLH80*, a gene from *E. breviscapus* that positively regulates flavonoid synthesis, was previously characterized. However, it is unclear whether *EbbHLH80* increases flavonoid accumulation, which affects salt tolerance. The function of *EbbHLH80* in transgenic tobacco seeds was identified by phylogenetic analysis and metabolome-transcriptome analysis. We investigated the role of *EbbHLH80* in salt stress response. Our results showed that the expression of *EbbHLH80* increased following salt treatment. Integrating the metabolome and transcriptome analysis of *EbbHLH80*-OE and Yunyan 87 (WT) seeds, we identified several genes and metabolites related to flavonoid biosynthesis and salt stress. Moreover, *EbbHLH80*-OE plants displayed higher salt tolerance than wild-type plants during seed germination and seedling growth. After salt treatment, transgenic tobacco had significantly lower levels of reactive oxygen species (ROS) than WT, with enhanced levels of antioxidant enzyme expression. Altogether, our results demonstrated that *EbbHLH80* might be a positive regulator, promoting salt tolerance by modulating ROS scavenging and increasing stress-responsive genes.

## 1. Introduction

Salt stress induces both osmotic and toxicity stresses in plants, which lead to growth inhibition, developmental changes, metabolic adaptations, and ion sequestration [1]. Salt stress triggers rapid production of ROS molecules, such as hydrogen peroxide, singlet oxygen, superoxide, and hydroxyl radicals, leading to oxidative damage to plants [2,3,4,5]. Previous studies have shown that transcription factors play a critical role in plant adaptation to abiotic stresses [6].

Several eukaryotes, from yeast to plants and animals, possess basic helix–loop–helix (bHLH) proteins, which belong to a large transcription factor (TF) family. This protein contains a region of 60 amino acids, which is divided into two functional domains [7]. *bHLH* transcription factors regulate plant growth and development, metabolism, and stress responses. Therefore, transcription factors act as molecular switches in plants while they respond to abiotic stress [8,9]. For example, *bHLH106* has been shown to be associated with salt tolerance in *Arabidopsis* [10]. *AtbHLH112* increased the expression of the *AtPOD* and *AtSOD* genes, while simultaneously reducing the expression of the *P5CDH* and *ProDH* genes, thus enhancing salt resistance in *Arabidopsis* [11]. *ThbHLH1* gene has been shown to increase peroxidase (POD) and superoxide dismutase (SOD) activities in response to high salt induction in *Tamarix hispida* [12]. Meanwhile, bHLHs may also be involved in the regulation of secondary metabolites that contribute to resistance to salinity. Salt stress activates *AtMYC2* via a mitogen-activated protein kinase (MAPK) cascade. *AtMYC2* can then bind to the promoter of *P5CS1*, thereby regulating proline synthesis and salt tolerance [13]. Additionally, *AtbHLH122* inhibited *CYP707A3* expression under NaCl stress [11]. Moreover, *EcbHLH57* can enhance tobacco resistance to salt stress by increasing the expression of stress responsive genes [14]. It appears that *OsbHLH068* has a similar effect on salt stress regulation as *OsbHLH112* that heterogenic overexpression of *OsbHLH068* can reduce salt-induced accumulation of H_2_O_2_ in *Arabidopsis* [15].

Plants produce a wide range of secondary metabolites with diverse chemical and physical properties. Plant secondary metabolites play a crucial role in their growth, development, and immunity [16]. The flavonoid pathway is usually initiated by physiological and environmental fluctuations as a protection against oxidative stresses, resulting from pathogen infections, UV, drought, salt, and nutrient deficiency, etc. [17]. Furthermore, flavonoids exhibit strong biological activity and significant antioxidant properties [18,19] and are involved in the protection of plants against UV radiation, salt, heat, and drought. Plants can resist biotic and abiotic stresses with flavonoids, such as flavonols and anthocyanins. Several studies have shown that the overexpression of *MYB12* confers resistance to insect attack in tobacco, and tolerance to salt and drought in Arabidopsis [20,21].

In a previous study, we characterized *EbbHLH80* in *Erigeron breviscapus*, which positively regulates flavonoid biosynthesis [22]. However, it is still unclear whether the increased flavonoid content was responsible for improving stress tolerance. With integrative metabolome-transcriptome analysis, we identified metabolites and genes related to flavonoid biosynthesis and salt stress in seeds of *EbbHLH80*-OE and Yunyan87 (WT). Furthermore, a positive correlation was observed between flavonoid contents with expression level of *EbbHLH80*, while *EbbHLH80*-OE plants exhibited hypersensitive phenotypes to salt stress during seed germination and seedling growth, compared to wild-type plants. *EbbHLH80*-OE modulated ROS scavenging, flavonoid biosynthesis, and stress-responsive gene expression in tobacco to enhance salt tolerance. Therefore, the above findings indicate that *EbbHLH80* plays a crucial role in salt stress tolerance. Furthermore, it could act as a critical available gene for genetic engineering breeding for plant salt resistance.

## 2. Results

### 2.1. Phylogenetic Analysis of EbbHLH80

Our previous studies reported that *EbbHLH80* increases the flavonoid contents in tobacco leaves [22]. Additionally, the protein sequences of highly homologous *Attt8* genes from various plants were conducted to further clarify the evolutionary relationship, the phylogenetic analysis showed that *Brassica napus TT8* (*BnTT8*, NP_ 001302903.1), *Brassica juncea TT8* (*BjTT8*, AIN41653.1), *Arabidopsis thaliana TT8* (*AtTT8*, CAC14865.1), and *EbbHLH80* were highly similar (Supplementary Figure S1). Previous studies showed that *TT8* acted as a crucial component of a well-conserved complex controlling flavonoid accumulation [23,24]. While we know that *EbbHLH80* plays a role in flavonoid biosynthesis, the role of *EbbHLH80* in seeds involved in responding to various stresses has not been reported. Thus, we investigated whether *EbbHLH80* is also involved in salt tolerance.

### 2.2. Differences in Metabolites with WT and EbbHLH80-OE Seeds

**Figure 1:**
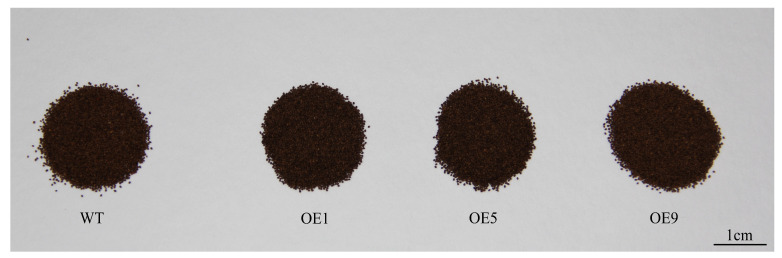
Phenotype diagram of tobacco seeds of WT and *EbbHLH80*-OE (OE1, OE5, and OE9). Bar = 1 cm.

**Figure 2:**
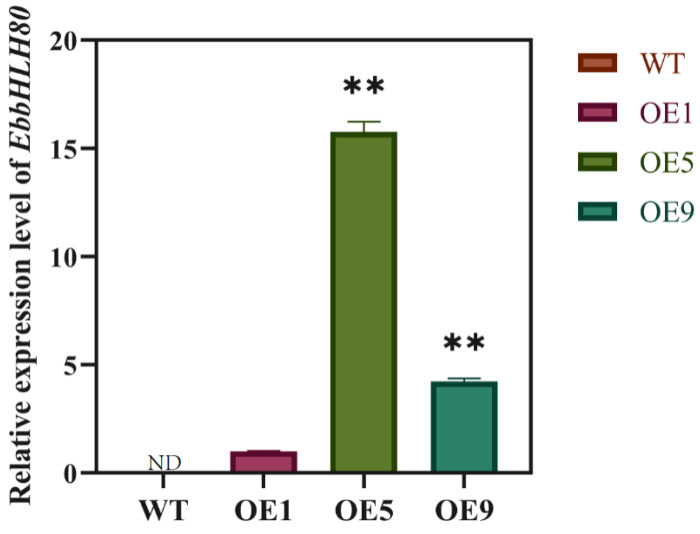
Transcript levels of *EbbHLH80* in WT and *EbbHLH80*-OE (OE1, OE5, and OE9) seeds. Error bars are standard errors (** *p* < 0.01).

The phenotypes of *EbbHLH80*-OE (OE1, OE5, and OE9) and WT seeds showed no significant differences (Figure 1). To evaluate the effects of *EbbHLH80* in tobacco seeds, the seeds of overexpression of *EbbHLH80* in the tobacco were confirmed by RT-qPCR analysis (Figure 2). The result confirmed that the transcript levels of *EbbHLH80* were significantly higher in OE5 than in OE1 and OE9. Subsequently, to further explore the changes in the metabolic levels in seeds of *EbbHLH80*-OE tobaccos, OE5 was chosen and used for metabolite analysis.

**Figure 3:**
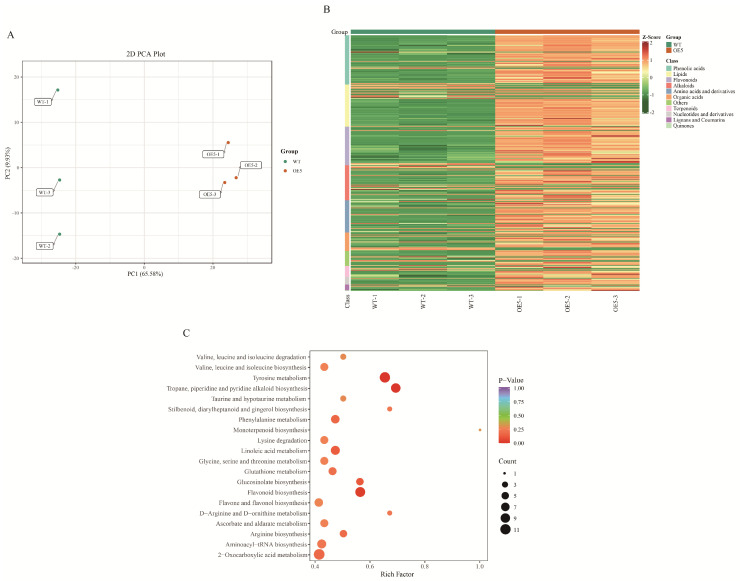
Metabolome analysis of WT and *EbbHLH80*-OE seeds. (**A**) PCA analysis of six samples. (**B**) Clustered heatmap of differential metabolites in WT and *EbbHLH80*-OE seeds. (**C**) KEGG enrichment plots of differentially accumulated metabolites in WT and *EbbHLH80*-OE seeds.

The metabolites of samples were analyzed by principal component analysis (PCA) to illustrate significant differences in metabolites between WT and OE5 seeds and were separated in the PCA score plots with 65.58% of PC1 and 9.93% of PC2 (Figure 3A). We obtained a total of 1136 metabolites, of which 301 were differentially accumulated metabolites (DAMs), including 301 upregulated-accumulated metabolites and 57 downregulated-accumulated metabolites (Supplementary Table S2). These metabolites were classified into 11 classes. The top 4 major classes of DAMs were phenolic acids (19.27%), lipids (16.48%), flavonoids (15.08%), and alkaloids (13.69%), respectively. To further elucidate the differences in metabolite accumulation in the seeds of WT and OE5, hierarchical clustering analysis was performed on all the DAMs of the obtained comparison pairs, and the relative quantitative values of the DAMs were normalized and clustered (Figure 3B). The results showed that the metabolites changed significantly between the seeds of WT and OE5.

Annotation of the metabolites was performed using the KEGG pathway database. The results identified that the DAMs in KEGG terms were principally related to flavonoid biosynthesis (Figure 3C), indicating that flavonoid accumulation was much higher in seeds of OE5 than those in WT. These results suggested that the function of *EbbHLH80* not only affect the flavonoid biosynthesis in leaves, but also in seeds. Thus, we speculated that DAMs in flavonoid biosynthesis might be initiated by overexpressing *EbbHLH80*.

### 2.3. Transcriptome Profiling of WT and EbbHLH80-OE

To understand the molecular basis of the metabolic differences between WT and *EbbHLH80*-OE, transcriptome sequencing was performed using the seeds of WT and OE5, with three biological replicates for each group. Data filtering produced an average of 6.69 Gb data for each sample. The Q20 value was in the range of 96.82–98.10%; the Q30 value was in the range of 91.55–94.14%; and the GC content was in the range of 43.69–44.45%. In general, the transcriptome data indicated that the sequencing quality was high, and the data obtained by sequencing are accurate and reliable.

**Figure 4:**
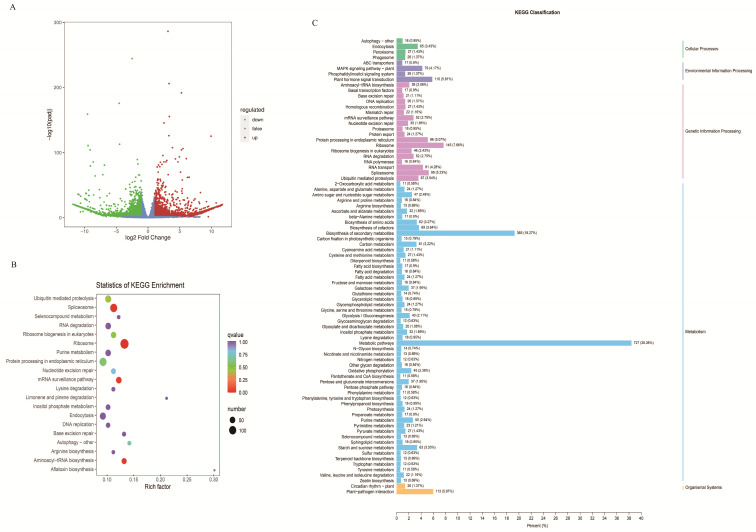
Transcriptome analysis of WT and *EbbHLH80*-OE. (**A**) Volcano plot of differentially expressed genes in WT and *EbbHLH80*-OE. The abscissa represents the gene expression fold change, and the ordinate represents the significance level of the differential genes. (**B**) GO classification of differentially expressed genes in WT and *EbbHLH80*-OE seeds. The abscissa represents the GO item, and the ordinate represents the number of differential genes of the GO item. (**C**) KEGG classification histogram of differentially expressed genes in WT and *EbbHLH80*-OE seeds. The abscissa represents the ratio of the number of differential genes annotated to the pathway to the number of differential genes annotated, and the ordinate represents the name of the KEGG pathway.

A total of 6438 differentially expressed genes (DEGs; A false discovery rate (FDR) < 0.05 and log2fold change ≥|1|) were identified (Supplementary Table S3), of which 3326 were up-regulated and 3112, as down-regulated, as shown in the volcano plots (Figure 4A). These DEGs were significantly associated with 55 GO functions and 136 KEGG pathways (Figure 4B,C). KEGG analysis showed that several metabolic processes, including plant hormone signal transduction, alpha-linolenic acid metabolism, phenylpropanoid biosynthesis, flavonoid biosynthesis, and flavone and flavanol biosynthesis were significantly enriched in DEGs (Figure 4C). This indicates that *EbbHLH80* might affect more genes that can be related to secondary metabolites biosynthesis in seeds.

### 2.4. EbbHLH80 Positively Regulates Tobacco Tolerance to Salt Stress

In plants, flavonoids are antioxidative agents that scavenge reactive oxygen species (ROS). Flavonoids inhibit ROS-generating enzymes, thereby preventing ROS production. Flavonoids are considered secondary antioxidant systems, since they complement other ROS scavenging systems that are reduced in activity. Our results showed that *EbbHLH80* could increase the accumulation of flavonoids in transgenic tobacco seeds; for example, the content of kaempferol increased 12.94-fold compared with that in WT, suggesting that these flavonoids contribute to the improvement of antioxidant capacity, thereby exhibiting a greater increase in responding salt stress in transgenic tobacco plants. Additionally, transcriptome analysis showed that some salt resistance-related genes were up-regulated. Therefore, we speculate that the impact of overexpressing *EbbHLH80* on salt tolerance is primarily from the increasing flavonoids accumulation, which improves the resistance of tobacco to salinity.

**Figure 5:**
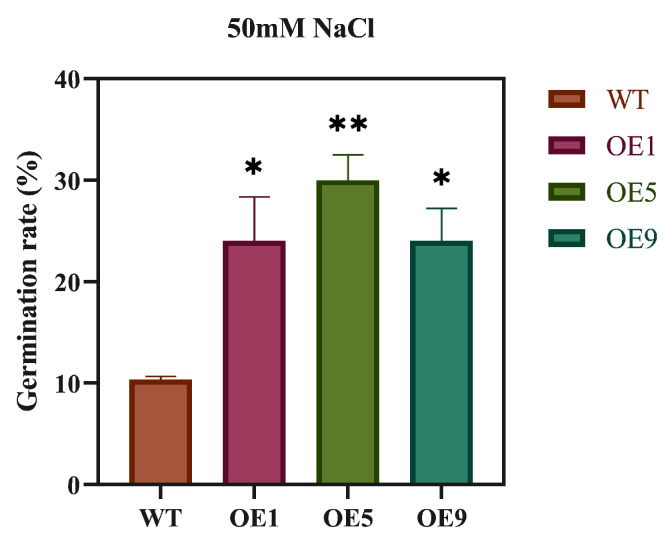
Differences in seed germination rates between WT and *EbbHLH80*-OE (OE1, OE5, and OE9) after 7 days under 50 mM NaCl treatment. Error bars are standard errors (** *p* < 0.01; * *p* < 0.05).

**Figure 6:**
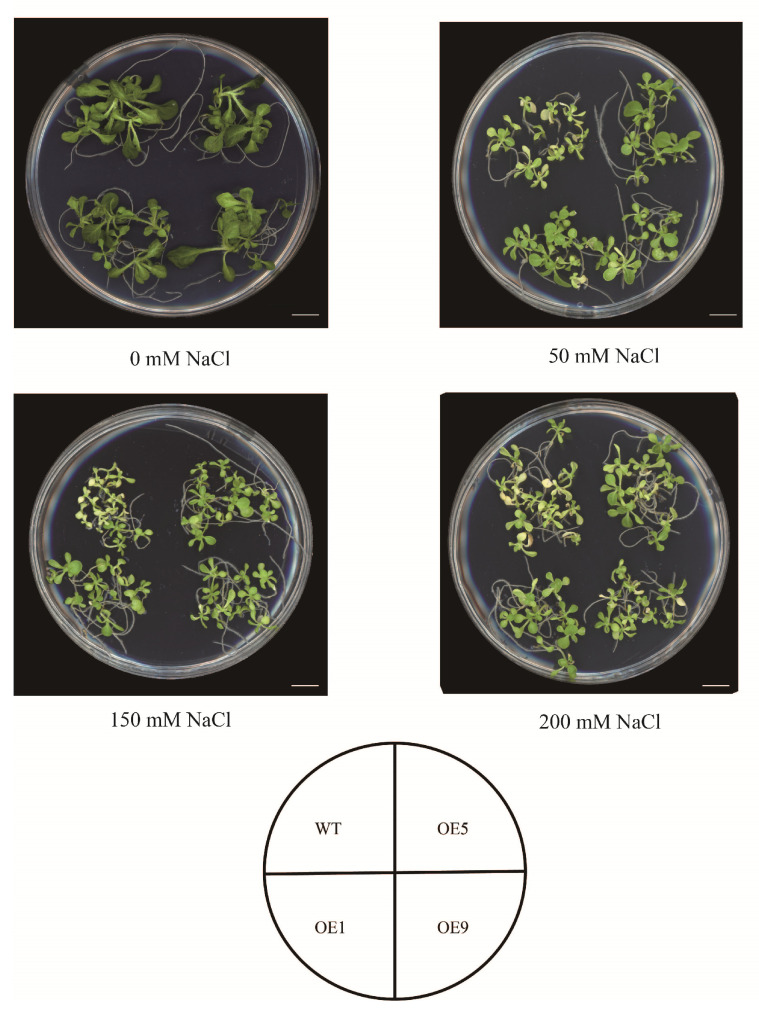
*EbbHLH80* increases salt tolerance in transgenic tobacco. Under salt stress, plant growth was significantly inhibited. Growth performance of WT and *EbbHLH80*-OE tobacco after treatment with 0 mM, 50 mM, 100 mM, 150 mM NaCl for 4 weeks. Scale bar = 1 cm.

To evaluate whether *EbbHLH80* could improve salt tolerance in transgenic plants, the response to salt stress was analyzed by measuring the germination rate of seeds from WT and *EbbHLH80*-OE after NaCl treatment (0, 50 mM) for 7 days. The results indicated that the germination rate of *EbbHLH80*-OE (OE1, OE5, and OE9) reached a substantially higher level than those in WT, while their response to salt stress, which was approximately 2.32-fold, 2.90-fold, and 2.32-fold higher, respectively (Figure 5). To demonstrate that salt stress would have an effect on the tobacco seedling phenotype of *EbbHLH80*-OE, tobacco seedlings were treated using different concentration of NaCl (0, 50, 100, 150 mM) for four weeks, respectively (Figure 6). It was observed that high concentrations of NaCl significantly inhibited the growth of tobacco and turned the leaves yellow. However, compared to wild-type tobacco, transgenic tobacco showed excellent growth conditions. We found that no significant difference was observed between the wild-type (WT) and *EbbHLH80*-OE lines (OE1, OE5, and OE9) when grown under normal conditions.

**Figure 7:**
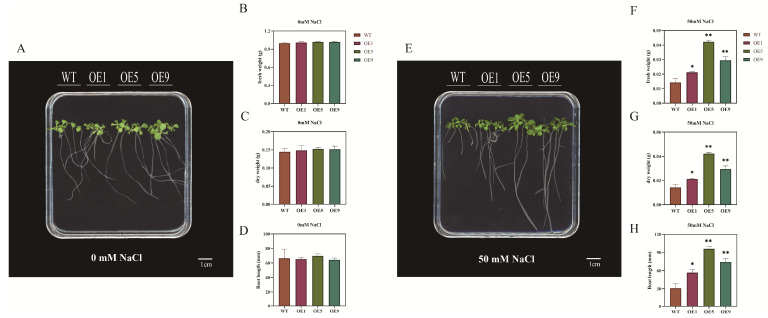
Seedling growth of WT and *EbbHLH80*-OE seedlings under salt stress. (**A**,**E**) Morphology of WT and *EbbHLH80*-OE seedlings (OE1, OE5, and OE9) under control treatment. Ten-day-old seedlings grown on ½ MS were transferred to MS medium with 0 mM NaCl and 50 mM NaCl treatment for 3 weeks. *N* = 4, Bar = 1 cm; (**B**–**D**,**F**–**H**) Quantitative comparison of fresh weight, dry weight, and root length of WT and *EbbHLH80*-OE seedlings (OE1, OE5, and OE9) under 0 and 50 mM NaCl treatment for 3 weeks. Error bars are standard errors (** *p* < 0.01; * *p* < 0.05).

To investigate whether salt stress could cause the root length to be affected, we examined the root length growth of WT and *EbbHLH80*-OE on 50 mM NaCl medium. Root length of WT seedlings was more severely inhibited than that of *EbbHLH80*-OE on MS plates supplemented with 50 mM NaCl (Figure 6), while *EbbHLH80*-OE tobacco showed longer roots (Figure 7A,E). Salt treatment reduced tobacco seedling growth parameters regardless of *EbbHLH80* expression changes. However, a less significant inhibition was observed in *EbbHLH80*-OE plants. Furthermore, after 3 weeks of salt treatment, *EbbHLH80*-OE plants showed greener leaves as compared to WT plants. To further explain the stress phenomenon, the fresh weight, dry weight, and root length were measured on tobacco plants. The results showed a higher level under 50 mM salt stress than those of the WT plants, indicating that *EbbHLH80* suppresses NaCl-induced growth (Figure 7B–D,F–H). Moreover, WT plants displayed a slower germination rate than *EbbHLH80*-OE plants in the presence of 50 mM NaCl. These results confirm that salt tolerance was significantly higher in *EbbHLH80*-OE (OE1, OE5, and OE9) than in WT.

### 2.5. EbbHLH80 Reduced the Damage Caused by Salt Stress in Transgenic Tobacco Seedlings

**Figure 8:**
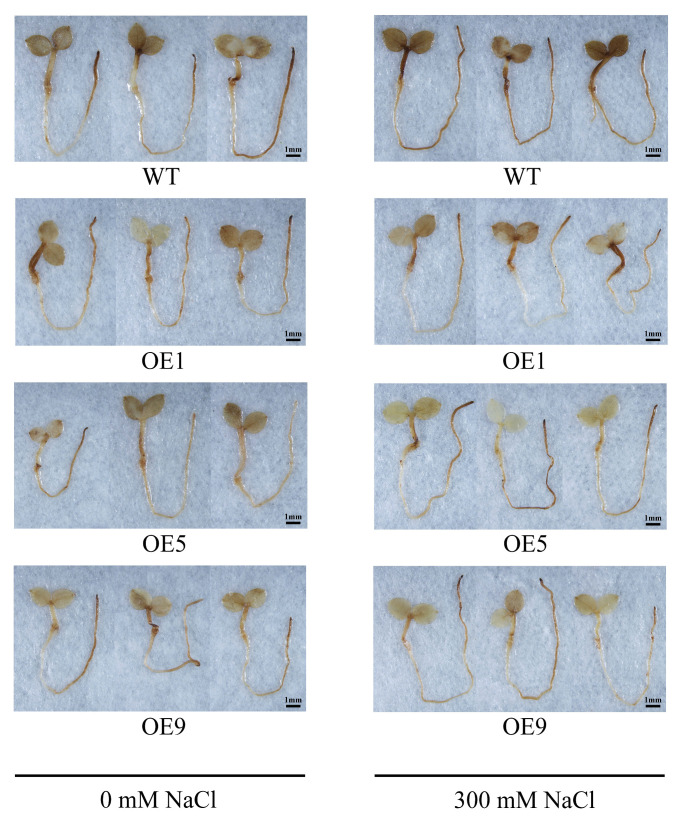
*EbbHLH80*-OE alleviates H_2_O_2_ accumulation in transgenic tobacco under salt stress. Histochemical localization of H_2_O_2_ accumulation in WT and *EbbHLH80*-OE seedlings with 0 mM NaCl and 300 mM NaCl visualized using DAB staining. Three replicates per treatment setup. Scale bar = 1 mm.

Reactive oxygen species (ROS) are produced by plants under unfavorable conditions [25,26]. ROS hyperaccumulation causes endogenous stress that damages plant growth and development [27]. The DAB staining was used to assess the amount of hydrogen peroxide (H_2_O_2_) released under salt stress, showing darker leaves in the salt treatment group than in the control group. It has been shown that plants under salt stress produce a large amount of H_2_O_2_, which can cause serious damage to them. To further address the role of *EbbHLH80* in salt stress response, all lines were transferred on 1/2 MS medium plates containing 300 mM NaCl and grown for 1 day. Salt stress directly reflects the degree of plant oxidation, exhibiting the extent of plant damage via H_2_O_2_. *EbbHLH80*-OE and WT seedlings accumulated H_2_O_2_ in different patterns after 300 mM NaCl treatment. The DAB staining showed a large accumulation of H_2_O_2_ in WT seedlings, whereas H_2_O_2_ accumulated less in OE1, OE5, and OE9 seedlings (Figure 8). These results suggest that *EbbHLH80* reduces the damage caused by salt stress in transgenic tobacco seedlings, indicating that *EbbHLH80* negatively responds to NaCl-induced cell death.

### 2.6. Expression Analysis of Genes Involved in ROS and Flavonoid Biosynthesis

**Figure 9:**
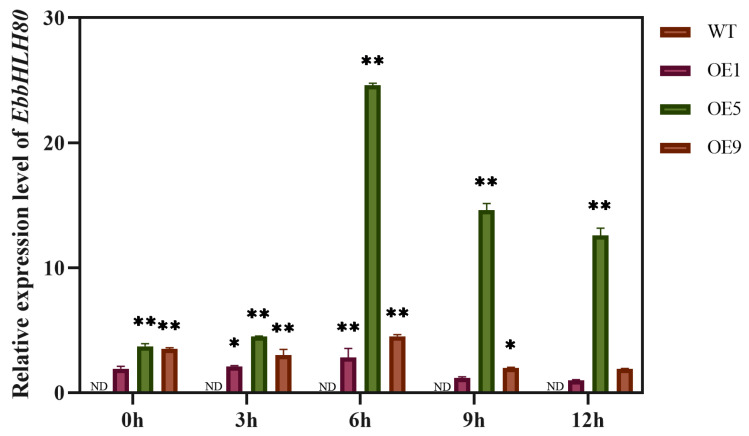
Expression pattern analysis of the *EbbHLH80*-OE in transgenic tobacco. The results were expressed as relative values with respect to 12 h of OE1, which were set to 1.0. Error bars are standard errors (** *p* < 0.01; * *p* < 0.05).

To illustrate that the expression of *EbbHLH80* will be affected under salt stress, we examined the expression of *EbbHLH80* by using quantitative real-time PCR (RT-qPCR). *EbbHLH80* transcript level was induced in 2-week-old WT and *EbbHLH80*-OE line seedlings after the treatment of 50 mM NaCl (for 1 h, 3 h, 6 h, 9 h, and 12 h), and reached the highest peak after 6 h, decreasing thereafter (Figure 9). The results showed that the expression level of *EbbHLH80* in transgenic tobacco seedlings was the highest at 6 h under 50 mM NaCl treatment (Figure 9).

**Figure 10:**
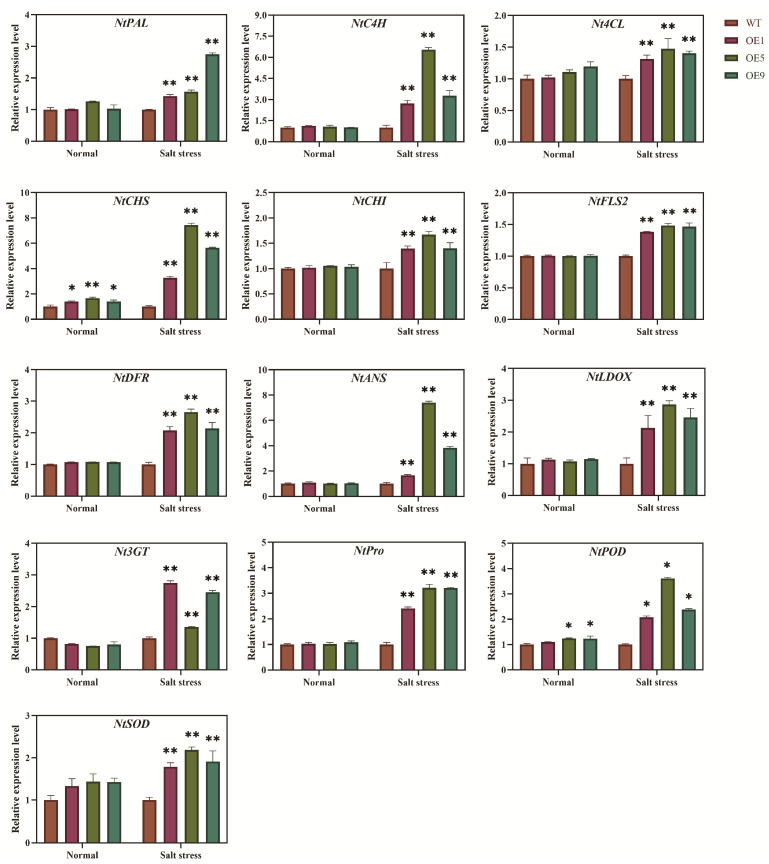
Relative expression level of the *EbbHLH80*-OE related genes in the transgenic tobacco under salt stress. Transgenic tobacco and WT seedlings were placed in a cultivation chamber and treated with 300 mM NaCl for 1 week to analyze the genes expression. The Nicotiana tabacum actin gene was used as an internal control. Mean values and standard errors were obtained from three biological and six technical replicates. The one-way analysis of variance was used for statistical analysis (** *p* < 0.01; * *p* < 0.05).

To further illustrate that *EbbHLH80*-OE can affect the changes of the expression amounts of some genes in transgenic tobacco seeds, we further analyzed the expression levels of genes related to salt stress, flavonoid biosynthesis, and ROS scavenging. Our results showed that after 300 mM NaCl treatment for 24 h, the expression levels of some genes involved in the flavonoid biosynthetic pathway were significantly higher in the tobacco seedlings of *EbbHLH80*-OE than those in WT, indicating that the expression patterns of these genes were associated with the regulation of *EbbHLH80* under salt stress, which was consistent with our previous findings. In addition, the expressions of genes related to *POD*, *SOD*, and proline that we found in our transcriptome data were also significantly up-regulated under salt stress, and this result indicated that *EbbHLH80* played a role in salt tolerance of transgenic tobacco. These results showed that the expression levels of genes involved in flavonoid biosynthesis and reactive oxygen species (ROS) system were significantly higher in transgenic tobacco compared with WT (Figure 10), and this finding was consistent with the transcriptome data, demonstrating the reliability of the transcriptome data.

## 3. Discussion

High salt stress is a major abiotic stress that inhibits plant growth, caused by ionic and osmotic stressors eventually and affecting crop production. Therefore, salt-tolerance in plants is a complex trait, acquired through the functions of multiple genes. Plant stress tolerance can be enhanced by utilizing stress-responsive transcription factors that regulate a wide range of downstream genes [28]. Several transcriptional factors have been identified in the response to salt stress in plants, including NAC, WRKY, MYB, AP2/ERF, and bHLH [29,30,31,32,33,34]. The *NAC* TF family, among the different TF families, has been receiving a lot of attention because of its functional diversity [35,36]. A previous study found that one of the *NAC* TFs, *PgNAC21*, is important for regulating salt tolerance in plants [37]. It has been found that MYB proteins contribute to plant salt tolerance. Salt tolerance is modulated by *MYB49* through the formation of cuticles and antioxidant defenses [38]. Furthermore, *MYB12* can also induce the expression of ABA biosynthesis genes, thereby enhancing salt tolerance in plants [23]. *MYB12* overexpression upregulates gene expression associated with flavonoid biosynthesis and ROS scavenging, as well as genes involved in ABA and proline biosynthesis, thus conferring salt tolerance [23,39]. *Rd29A* and *AP2*/*ERF* family transcription factor *DREB2A*, are upregulated by salt treatment, both of which contain DRE in their promoters [40].

In previous studies, bHLHs have been found to regulate plant adaptation to stress across several pathways, including resistance to mechanical damage, drought, high salt, oxidative stress, low temperature stress, heavy metal stress, iron deficiency, and osmotic stress, according to previous research [15,41]. The main environmental factor limiting plant growth and productivity is salt stress. The effects of salt stress can include ionic stress, osmotic stress, and secondary stresses, especially oxidative stress [42,43]. These studies suggested that transcription factor regulation might affect plant salt tolerance. Herein, it was demonstrated that the over-expression of *EbbHLH80* contributed to a considerable improvement of salt-stress tolerance. *EbbHLH80* reduced cell death of transgenic tobacco under salt stress. Similarly, some *bHLH* TFs have a regulatory effect under salt stress. Plant cells simultaneously undergo osmotic stress, ion toxicity, and oxidative stress under salt stress [42]. *BnTT8* and *AtTT8* have been demonstrated to be involved in salt tolerance [24,44]. *AtNIG1* is also a *bHLH* transcription factor that has been shown to play a role in salt stress signaling pathways in Arabidopsis [45]. Additionally, *OsbHLH062* regulates ion transport genes, such as *OsHAK21*, and modulates Jasmonic acid (JA) signaling pathways in order to confer salt tolerance to plants [46].

The antioxidant properties of flavonoids are generally responsible for enhancing stress-resistance. Abiotic stresses, such as low temperatures, drought, and salt stress, result in a series of physiological and biochemical changes in plant cells, primarily causing a decrease in photosynthesis efficiency and generating reactive oxygen radicals to make serious cell damage [47]. Oxidative stress activates flavonoid synthesis, protecting cells from oxidative damage and preventing reactive oxygen species [48]. Moreover, *ZmSRO1e* prevents anthocyanins from hyperaccumulating, helping to maintain ROS homeostasis and balance between growth and abiotic stress [49]. Moreover, *AmDEL* gene overexpression increased the expression of genes related to flavonoids, proline, and reactive oxygen scavenging under salt and drought stress [50]. Therefore, flavonoids are not only involved in removing reactive oxygen species in plants, but also act as signaling molecules [51]. We found that several flavonoid synthesis genes were upregulated in transgenic tobacco seeds, suggesting that these genes may be involved in salt tolerance mediated by *EbbHLH80*. To determine whether *EbbHLH80* mediates salt tolerance by up-regulation of these genes, *PAL*, *C4H*, *4CL*, *CHS*, *CHI*, *FLS2*, *DFR*, and *ANS* were randomly selected for salt tolerance study. The results showed that these genes were all positively correlated with salt stress tolerance (Figure 9). Together, the results indicated that flavonoid biosynthesis-related genes played a role in salt stress, and *EbbHLH80* improved salt tolerance by promoting their expression. ROS are produced by plants while they encounter adverse environments. In plants, ROS play a dual role, acting as signaling molecules at low levels and causing oxidative stress at high levels [52]. Therefore, the regulation of ROS at appropriate levels is vital for plants under abiotic stress [53].

Salt tolerance is mediated by *bHLH* TFs regulating ROS balance through direct regulation of peroxidase genes. *AtbHLH92* enhanced *Arabidopsis* tolerance to osmotic and salt stresses by partially regulating ABA and SOS2 [54]. In addition, *BvbHLH92* is expressed in both roots and leaves of beets when exposed to salt stress [54]. We found that *EbbHLH80* could induce the expression of genes including *SOD*, *POD*, and *Pro* (Figure 9), accompanied by decreased ROS levels (Figure 8). Furthermore, GO classification analysis showed that genes involved in antioxidant activity were highly expressed. Together, these findings suggest that *EbbHLH80* may induce ROS scavenging-related genes, including *SODs* and *PODs*, resulting in increased POD and SOD activity and subsequently improved salt tolerance.

## 4. Materials and Methods

### 4.1. Plant Materials and Growth Conditions

*Nicotiana tabacum* cv. Yunyan87 (Y87, WT) and *E. breviscapus* obtained from the Yunnan Agricultural University (Kunming, China) were used in this study. Yunyan87 (Y87, WT) and *EbbHLH80*-OE lines were grown at greenhouse in Yunnan Agricultural University (Kunming, China). Fully mature seeds were collected from normally grown T2 generation *EbbHLH80*-OE tobacco for the following experiments. For exogenous addition of NaCl on 1/2 MS medium (Murashige and Skoog medium, with sugar, 0.7% agar, pH 5.7) experiments, tobacco seeds were disinfected with 75% ethanol for 6 min and rinsed six times with sterile water. After seed germination, 10-day-old seedlings were transferred to MS medium supplemented with NaCl to observe the growth phenotype. Surface-sterilized WT and *EbbHLH80*-OE seeds were grown in 1/2 MS medium at 4 °C for 2 days before placing them in tissue culture at 24 °C for 16-h-light/8-h-dark cycle. The subsequent addition of 300 mM NaCl was used in RT-qPCR experimental material.

### 4.2. Metabolite Extraction and Analysis

The WT and *EbbHLH80*-OE (OE5) tobacco seeds metabolites were detected by UPLC (SHIMADZU Nexera X2) and MS/MS (Applied Biosystems 4500 QTRAP). According to the method with modifications [55]. WT and OE line seeds are vacuum freeze-dried in a freeze dryer (Scientz-100F) and then ground into powder with a grinder (MM 400, Retsch, Haan, Germany). Dissolve 100 mg of sample in 1.2 mL 70% in the chromatographic sample bottle for UPLC-MS/MS analysis. For peak alignment, pickup, and quantification of each metabolite by UHPLC-MS/MS.

HPLC: column, Agilent SB-C18, 1.8 µm, 2.1 mm × 100 mm; mobile phase: phase A is ultrapure water (add 0.1% formic acid), and phase B is acetonitrile (add 0.1% formic acid); elution gradient: 5% phase proportion at 0.00 min, linear increase of phase proportion to 95% in 9.00 min and maintenance at 95% for 1 min, 10.00–11.10 min, 5% phase proportion drop in B, and equilibration at 5% for 14 min; flow rate 0.35 mL/min; temperature, 40 °C; injection volume: 4 mL.

ESI source operating parameters were as follows: ion source, turbo spray; source temperature 550 °C; ion spray voltage (IS) 5500 V (positive ion mode)/−4500 V (negative ion mode); the ion source gas I (GSI), gas II (GSII), and curtain gas (CUR) were set to 50, 60, and 25.0 psi, respectively, and the collision induced ionization parameter was set to high. A specific set of MRM ion pairs was monitored in each epoch based on the metabolites eluted within each epoch.

### 4.3. RNA Extraction and Sequencing

Frozen seeds (WT and *EbbHLH80*-OE) were ground into powder using liquid nitrogen. Total RNA was extracted from frozen seeds using the HiPure HP Plant RNA Mini Kit (Magen, Guangzhou, China). High quality RNA was used to synthesize cDNA libraries. After completion of cDNA library construction, the library quality was checked by initial quantification with Qubit2.0 and insert size of the library by Agilent 2100, followed by accurate quantification by Q-PCR (effective concentration of the library is >2 nM). The fragments were sequenced on an Illumina HiSeq 4000 platform.

### 4.4. RNA Sequencing (RNA-Seq) Differential Gene Expression Analysis

DESeq2 was used to examine gene expression differences between two groups samples. False discovery rate (FDR) values were then corrected for multiple hypothesis testing with the Benjamini-Hochberg method. Differential genes were filtered with a log2fold change ≥ |1| and FDR < 0.05.

### 4.5. Determination of H_2_O_2_ Content in Seedlings after Salt Stress Treatment

Seedlings grown on 1/2 MS medium (0.7% agar) for 2 weeks were treated with 300 mM NaCl for 1 h, control is treated with water of the same volume. Seedlings of WT and *EbbHLH80*-OE lines were soaked in 1 mg/mL DAB staining solution, staining at room temperature in the dark for 6 h. After rinsing in pure water, the stained seedlings were decolorized by immersion in 95% ethanol at 40 °C for 12 h and were photographed after the decolorization ended by washing well and placing them in DAB sample preserving solution for 30 min (Solarbio, Beijing, China).

### 4.6. RT-qPCR Analysis of Gene Expression

RT-qPCR was used to verify the feasibility of the overexpression experiments and the credibility of the transcriptome data. Seeds for analyzing *EbbHLH80* expression patterns and seedlings after salt stress were frozen with liquid nitrogen. WT and OE line of RNA was isolated following the instructions of manufacturer (Magen, Guangzhou, China) and the cDNA was synthesized using a PrimeScript™ RT reagent Kit with gDNA Eraser (Takara, Beijing, China). RT-qPCR was performed in the ChamQ SYBR qPCR Master Mix (Vazyme, Nanjing, China), with six biological replicates. *NtActin* (XM_016628756) was used as an internal reference gene to calculate the difference in specific expression of the target gene. Specific primer pairs for each gene (Supplementary Table S1) were designed by Primer3 (v. 0.4.0).

### 4.7. Statistical Analysis

All data presented in this manuscript were collected from more than three independent replicates and were statistically analyzed by one-way analysis of variance. Standard errors were calculated for the means and significant differences determined at the significance levels of *p* ≤ 0.05 and *p* ≤ 0.01.

## 5. Conclusions

Overall, a bHLH family gene, *EbbHLH80*, has been identified from *E. breviscapus* in a previous study. *EbbHLH80* was induced by salt-stress treatment. The overexpression of *EbbHLH80* resulted in enhanced resistance to salt stress in tobacco. Furthermore, transgenic plants displayed higher expression levels of three antioxidant enzymes (*SOD*, *POD*, and *Pro*), causing less ROS accumulation. Several flavonoid biosynthesis genes were also found to be up-regulated in the transgenic lines. In the future, *EbbHLH80* can act as a candidate gene to enhance the stress tolerance of *E. breviscapus* with genetic engineering techniques.

## Data Availability

All original transcriptome data were submitted to NCBI and available for download after 12-months (Accession Number: PRJNA923040 (*EbbHLH80*-OE, https://dataview.ncbi.nlm.nih.gov/object/PRJNA923040?reviewer=ln75feqsdisboch313ed80m2b2), PRJNA923042 (Yunyan 87, https://dataview.ncbi.nlm.nih.gov/object/PRJNA923042?reviewer=258v754pemp987qehvcfq4agr4). Dataset accessed on 1 February 2024. The dataset generated during and/or analyzed during the current study are available from the corresponding author on reasonable request.

## Supplementary Materials

The supporting information can be downloaded at: https://www.mdpi.com/article/10.3390/ijms241311080/s1.
