# Supplementary material for: EbbHLH80 Enhances Salt Responses by Up-Regulating Flavonoid Accumulation and Modulating ROS Levels
Source: Int J Mol Sci. 2023 Jul 4;24(13):11080. doi: 10.3390/ijms241311080 (PMC10341687; doi:10.3390/ijms241311080)
Supplement: Supplementary file 1 [file ijms-24-11080-s001.zip › Supplementary figure S1.pdf]

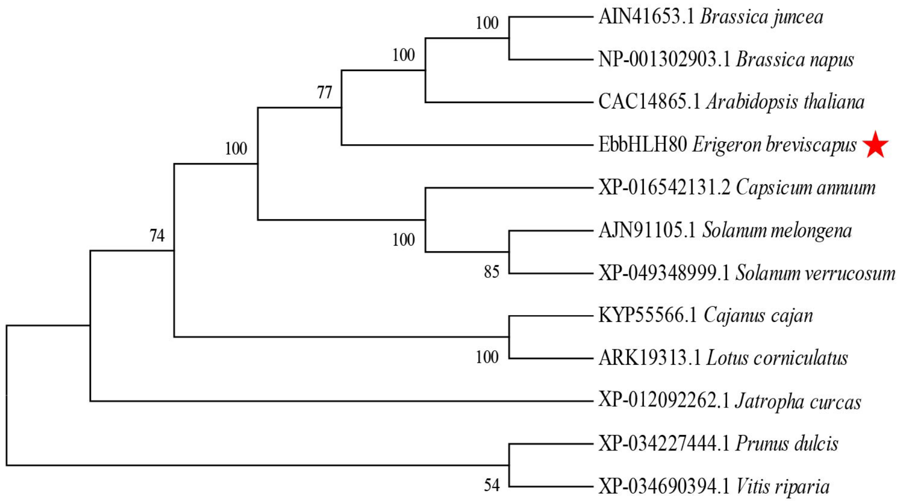

Figure S1 Phylogenetic tree of EbbHLH80 protein and related homologs in different species. Branch length is proportional to distance.
